# The intestinal neuro-immune axis: crosstalk between neurons, immune cells, and microbes

**DOI:** 10.1038/s41385-020-00368-1

**Published:** 2021-02-04

**Authors:** Amanda Jacobson, Daping Yang, Madeleine Vella, Isaac M. Chiu

**Affiliations:** Department of Immunology, Harvard Medical School, Boston, MA 02115 USA

## Abstract

The gastrointestinal tract is densely innervated by a complex network of neurons that coordinate critical physiological functions. Here, we summarize recent studies investigating the crosstalk between gut-innervating neurons, resident immune cells, and epithelial cells at homeostasis and during infection, food allergy, and inflammatory bowel disease. We introduce the neuroanatomy of the gastrointestinal tract, detailing gut-extrinsic neuron populations from the spinal cord and brain stem, and neurons of the intrinsic enteric nervous system. We highlight the roles these neurons play in regulating the functions of innate immune cells, adaptive immune cells, and intestinal epithelial cells. We discuss the consequences of such signaling for mucosal immunity. Finally, we discuss how the intestinal microbiota is integrated into the neuro-immune axis by tuning neuronal and immune interactions. Understanding the molecular events governing the intestinal neuro-immune signaling axes will enhance our knowledge of physiology and may provide novel therapeutic targets to treat inflammatory diseases.

## Introduction

### A brief history of neuroimmunology

Scientific exploration of the interaction between the nervous system and the immune system has old roots. This history has been well-documented in the context of pain and neurogenic inflammation. The inflammatory response was described two thousand years ago by Roman encyclopedist Aulus Cornelius Celsus (BC 25–AD 50), defined by the four cardinal signs of rubor (redness), tumor (swelling), calore (heat), and dolore (pain).^1^ Pain, an unpleasant sensation driven by the nervous system, is thus intrinsically linked to inflammation. In the last few hundred years, researchers began to demonstrate that the peripheral nervous system, including sensory and autonomic neurons, actively signal to the immune system and vasculature to drive inflammation.

Physiological experiments in the late 1800s laid the foundation, showing that the inflammatory response described by Celsus is mediated by peripheral nerves. For example, it was shown that denervation of skin led to changes in vasodilation that accompanies inflammation.^2,3^ Such experiments, combined with the developing understanding that neurons innervate peripheral barrier tissues in mammals, provided a foundation for future molecular studies.^4–6^ In the early 1900s, it was shown that neural activators such as mustard oil cause inflammation in a sensory neuron-dependent fashion.^7–9^ This was thought to occur through a local “axon reflex,” in which an initial stimulus activates nerves, leading to a spread of activation beyond the initial stimulus.^8^ This idea explains the observation that hallmarks of inflammation are often apparent in a larger area than the initial stimulus (i.e., injury, site of infection). Sir Thomas Lewis corroborated these findings and described a neuronal “triple response” mediated by the axon reflex (the wheal, flare, and local reddening) that describe vasodilation and edema we commonly associate with inflammation.^10^ He found that severing skin-innervating nerves led to a decreased flare response, indicating a neurogenic component of inflammation.

A re-emergence of work on this topic in the 1960s led to the characterization of neural mediators of inflammation, including the neuropeptides substance P (SP), calcitonin gene receptor peptide (CGRP), and others.^9,11–13^ Molecular and physiological studies found that CGRP and SP act on smooth muscle cells, endothelial cells and immune cells to drive vasodilation, edema, and tissue inflammation.

A key concept in neuroimmunology, in addition to local axonal reflexes, involves long-range sensori-autonomic reflexes where parasympathetic or sympathetic neurons play the major role in regulating immunity. A groundbreaking example of this concept, the “inflammatory reflex,” was first described by Kevin Tracey and colleagues in the early 21st century. This neuro-immune circuit involves the vagus nerve and its modulation of TNF-α production in the spleen during peripheral inflammation, including endotoxic shock and bacterial sepsis. In this reflex circuit, vagal sensory afferents detect inflammation, leading to brainstem and autonomic feedback through vagal efferents which ultimately lead to suppression of systemic TNF production. In the spleen, neuronal release of epinephrine and norepinephrine activate ChAT+ (choline acetyltransferase) T cells to produce acetylcholine, which in turn inhibits TNF-α production by splenic macrophages.^14–18^

The molecular mechanisms of bi-directional neuro-immune signaling is a major recent topic of research. Neurons express receptors for cytokines and lipid mediators released by immune cells, including canonical cytokines like TNF-α and IL-1β.^19–24^ Many immune mediators can functionally alter neuronal signaling, including sensitization of sensory neurons in the context of pain.^25,26^ Pattern-recognition receptors, canonically expressed by immune cells, are expressed by neurons, allowing simultaneous surveyance of luminal microbial signals by both systems.^27–30^ Immune and epithelial cells express cognate receptors for neurotransmitters and neuropeptides as well as neuronal mediators, laying the foundation for bidirectional communication.^27,28,31,32^ Taken together, the field of neuroimmunology has a long history, from the identification of local and systemic reflexes, and the identification of molecular mechanisms leading to neuro-immune crosstalk. The described studies and many others have renewed interest in this research area, birthing a renaissance in the study of neuroimmunology.

### Neuro-immune interactions in the intestine

The gastrointestinal immune system and nervous system have both evolved mechanisms to sense and rapidly respond to the dynamic intestinal environment. Many nerves appose local immune cells in the gastrointestinal mucosa to form neuron-immune cell units that can be reshaped by gut luminal nutrient-derived and microbe-derived cues.^30,33–35^ These units not only independently initiate corresponding reactions, but also communicate to form a neuro-immune axis which is modulated by the intestinal microbiota.^36–43^ It is becoming clear that these concerted signaling axes regulate gastrointestinal barrier function, immunity, and host protection. Despite this progress, the cellular circuits and molecular mechanisms mediating such interactions are only beginning to be understood and require further investigation.

In this review, we discuss recent studies advancing our understanding of neuro-immune crosstalk in the gastrointestinal tract specifically. Our review focuses on recent studies and does not cover all aspects of neuro-immunology. We recommend outstanding reviews for information on current topics in neuro-immunology from Tracey et al. and Veiga-Fernandes et al..^44–47^ We begin with a brief introduction of the neuroanatomy of the gut. Then, we discuss recent research dissecting neuron communication with innate immune cells, adaptive immune cells, and epithelial cells. To conclude, we examine how the gut microbiota tunes neuro-immune interactions, adding another layer of complexity to this crosstalk.

## Neuroanatomy of the gastrointestinal tract

**Fig. 1:**
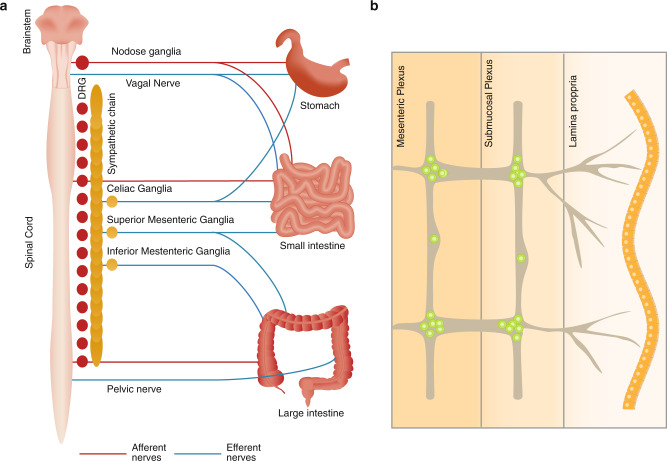
Neuroanatomy of the gastrointestinal tract. Left panel: Neuroanatomy of extrinsic innervation of the gastrointestinal tract. Extrinsic parasympathetic, sympathetic and sensory neurons originate in the brainstem and spinal cord. Sympathetic innervation through the sympathetic chain of the spinal cord occurs through the extrinsic celiac, and superior and inferior mesenteric ganglia. The distal colon is innervated by the pelvic nerves. Innervation from specific DRG and the sympathetic chain represent individual examples of how the intestine is innervated from these sources. With respect to the intestine, thoracic DRGs are primarily proximal-innervating, while lumbar DRGs are primarily distal-innervating. Right panel: Neuroanatomy of the enteric nervous system. Intrinsic enteric neurons and glial cells reside in the myenteric and submucosal plexus layers of the intestine and innervate all intestinal layers. Extrinsic neurons innervate this network to integrate signals from the brainstem and spinal cord with the intestine.

The gastrointestinal tract, anatomically composed of mesentery, serosa, muscularis, submucosa, lamina propria and epithelium, is innervated by multiple peripheral neuron populations which appose and coordinate responses with local immune cells.^33–35,44–49^ The nomenclature of gut-innervating neurons is based on whether the cell bodies are located outside or inside of the gastrointestinal tract. Enteric-associated neurons are generally classified as belonging to the intrinsic enteric nervous system (ENS). By contrast, gut-extrinsic neurons include somatosensory neurons and autonomic neurons whose cell bodies reside in peripheral sensory or autonomic ganglia, the spinal cord, or brainstem (Fig. 1).

Sympathetic neurons and parasympathetic autonomic neurons originate in the spinal cord and brainstem, respectively, and mediate signal transduction from the brain to the gut. Sympathetic neurons drive the body’s stress response, executing inhibitory intestinal functions including slowing of intestinal motility and secretion. Sympathetic neurons signal via catecholamines (dopamine, epinephrine, norepinephrine) to α- or β-adrenergic receptors. These neurons are subclassified based on their inhibitory function, including postganglionic vasoconstrictor neurons, secretion inhibitory neurons and motility inhibitory neurons.^44,50–53^ The distal colon and rectum are innervated by pelvic nerves, which have recently been shown to be sympathetic.^54^ In contrast, parasympathetic neurons mainly originate in the brainstem, innervating the gut through the efferent vagus nerve, and mediate gut physiology via the neurotransmitter acetylcholine (ACh) in driving motility, digestion, and secretory function.^44,48,55^ Vagal parasympathetic nerves innervate the GI tract in a rostrocaudal direction, with the highest density of innervation in the stomach and decreased innervation in the small intestine and colon.^22^ Both sympathetic and parasympathetic neurons form connections with the ENS in the myenteric plexus to form intricate local neural circuits.

Extrinsic gut-innervating sensory neurons residing within the nodose ganglia and dorsal root ganglia (DRG) transduce sensory signals from the gut to the brainstem and spinal cord, respectively.^34,42^ These neurons are pseudo-unipolar in nature, with a peripheral axon that terminates in the end-organ such as the gut, and a central axon that terminates in the CNS. These sensory neuron populations detect nutrients, mechanical stretch, luminal threats, and immune stimuli including cytokines. These neurons also signal within the gut by their release of neuropeptides such as CGRP and substance P from their peripheral nerve terminals.^34,56,57^ Vagal ganglia neurons mainly innervate the proximal small intestine, whereas DRG neurons innervate throughout the GI tract.^45,58,59^

Enteric neurons reside fully within the GI tract, and are organized into ganglionated networks encircling the intestinal tube and spatially categorized into two layers: the myenteric plexus, between the circular and longitudinal muscle layer, and the submucosal plexus in the submucosa (Fig. 1).^24,44,60^ These two plexii are closely interconnected by interneurons, motor neurons, and enteric glial cells. Together, they form reflex circuits that mediate peristalsis and secretory function. In addition, they also integrate signals from extrinsic sensory, parasympathetic, and sympathetic neurons to mediate gut physiology.^36^

Different branches of the extrinsic and intrinsic nervous systems can crosstalk with immune cells in the GI tract. Understanding their coordinated signaling with immune cells is of importance in mucosal immunology. In the following sections, we will review how distinct neurons communicate with resident innate and adaptive immune cells to regulate intestinal gut function at homeostasis and during disease.

## Neuronal crosstalk and innate immunity

Both the nervous system and innate immune system encode the ability to rapidly detect and respond to danger signals: for example, by Toll-like receptors for pathogen detection and ion channels that detect ATP and other damage-associated molecules.^44,48^ Considering this shared function, neuronal crosstalk with innate immune cells likely facilitates synergistic sensing of environmental cues and rapid adaptation to tissue injury and infection. We focus this section on neuronal communication with three gut-resident innate immune cells: macrophages, innate lymphoid cells (ILCs), and mast cells. While neurons have been shown to signal to other innate immune cells,^23,61–63^ these three intestinal neuro-immune interactions have garnered particular focus in recent years.

### Neuron–macrophage interactions

Gastrointestinal macrophages have been found to be critical for tissue homeostasis and host defense, with diverse phenotypic and functional specialization based on residence in particular intestinal tissue layers.^64,65^ Macrophages and nerves reside in close proximity in several intestinal layers at homeostasis and in a variety of disease contexts.^64,65^ Recent work has begun to show that neuronal signals play a critical role in macrophage function, and that macrophages regulate neuronal health during both homeostasis and in pathology.

**Fig. 2:**
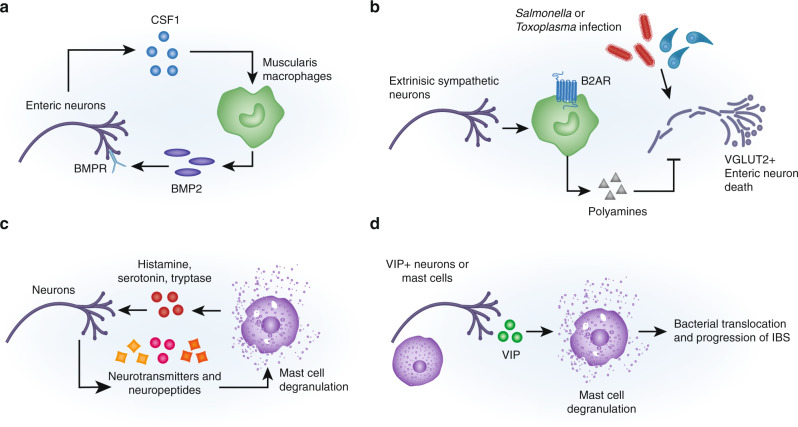
Neuronal crosstalk with intestinal macrophages and mast cells. **A** Muscularis Macrophages (MMs) release bone morphogenic protein 2 on enteric neurons mediates intestinal motility; in turn, enteric neurons release CSF1 to promote MM survival. **B** During infection, extrinsic sympathetic neuron release of norepinephrine stimulates polyamine synthesis which prevents enteric neuron inflammasome-mediated cell death. **C** Neurons and mast cells communicate bidirectionally. Neurons produce neuropeptides and hormones that trigger mast cell activation and degranulation; in turn, mast cells can produce histamine, serotonin and tryptase that can regulate neuronal function. **D** In IBS, production of VIP triggers mast cell degranulation which is critical for disease progression.

Initial mechanistic understanding^s^ of gastrointestinal bi-directional neuron-macrophage crosstalk resulted from characterization of a population of Muscularis Macrophages (MMs) in the *muscularis externa* layer which houses the ENS and circular and longitudinal smooth muscle.^66^ MHCII^+^Cd11b^+^CX_3_CR1^+^ MMs exhibit a tissue-protective phenotype characterized by expression of genes such as *Arg1*, *IL10, Mrc1, Cd163, and Retnla*, which is in contrast to lamina propria macrophages closer to the lumen that exhibit a pro-inflammatory phenotype.^67^ MMs are in close proximity to enteric ganglia and regulate homeostatic intestinal motility, as MM depletion results in abnormal muscle contraction and slower intestinal transit time (Fig. 2a). The mechanism underlying this process likely relies on MM production of the growth factor bone morphogenic protein 2 (BMP2); exogenous BMP2 is sufficient to rescue intestinal motility defects in MM-depleted mice. BMP2 signals through the bone morphogenic protein receptor (BMPR) on enteric neurons to orchestrate muscular contraction. In turn, enteric neurons promote MM maintenance through production of the macrophage survival factor CSF1 (also called M-CSF), which is essential for MM preservation in the tissue.^66^

Neuron–MM interactions also play an important role in neuronal protection during infection-induced inflammation and tissue destruction (Fig. 2b). After clearance of enteric bacterial or parasitic infections, inflammasome-mediated death of excitatory VGLUT2+ enteric neurons occurs in the muscularis, leading to long-lasting motility defects.^31^ MMs limit infection-induced enteric neuron loss through the generation of polyamines.

Sympathetic neurons that innervate the myenteric plexus maintain a tissue protective, M2-like phenotype in these macrophages. Transcriptomics of MMs indicates that these macrophages express high levels of the β2 adrenergic receptor (β_2_AR). Gut-extrinsic muscularis*-*innervating sympathetic neurons become activated during infection, showing c-fos expression, and signal by release of norepinephrine that acts through β_2_ARs on MMs, which in turn release polyamines that protect enteric neurons from cell death and pathology. Therefore, this sympathetic neuro-immune axis maintains homeostasis and neuroprotection in the gut.^31,67^

Intestinal neuron–macrophage interactions are beginning to be more broadly defined. A recent study showed that many MMs appear to be a subset of CX_3_CR1^+^ intestinal macrophages that are self-maintained by embryo-derived progenitors in the gut, rather than the majority of intestinal macrophages originating continuously from infiltrating bone marrow monocytes.^68^ Such self-maintaining macrophages reside not only within the muscularis, but also in the submucosa and juxtaposed to the vasculature, performing critical tissue support functions including maintaining neuronal health, adhesion, and angiogenesis; however, the genes characterizing this population (*Nova1*, *Chrm2*, and *Efr3b*) do not consistently overlap with those of MMs characterized in other studies.^67,68^ These macrophages are intimately associated with enteric neurons and mediate neuronal homeostasis. The depletion of self-maintaining macrophages results in caspase three-dependent enteric neuron apoptosis, altered neuron function including loss of neuron-evoked anion secretion, and slowing of intestinal motility.^68^

There are many important questions that remain about how macrophages and neurons interact in the intestine and what the consequences of these interactions are. While recent work has focused on macrophages in the muscularis and myenteric plexus, it is currently unclear if and how macrophages in other intestinal layers, including the lamina propria, communicate with neurons. This is very likely, considering that the submucosal plexus and lamina propria are also densely colonized by macrophages and innervated by intrinsic and extrinsic neurons.^65,69^ Future work is needed to characterize how specific neurons, including pain-mediating sensory neurons, enteric neurons, parasympathetic and sympathetic neurons, crosstalk with macrophages at homeostasis or during pathology. It would also be interesting to determine whether inflammatory bowel diseases (IBD), irritable bowel syndrome (IBS), or other gastrointestinal diseases show dysregulated crosstalk between neurons and macrophages.

### Neuron–mast cell interactions

Mast cells are located in the mucosal and submucosal layers throughout the GI tract and display close anatomical association with the nerve endings from sensory and autonomic neurons, forming an intimate and bidirectional communication (Fig. 2c). Mast cells express an abundant array of receptors, like IgE receptor FCeR1 and TLRs, which upon activation induce rapid degranulation to release inflammatory mediators like histamine, serotonin, and tryptase, which sensitize the threshold of firing for sensory neurons driving visceral pain. These neurons express histamine receptors, serotonin receptors and proteinase activated receptor 2.^70^ In turn, sensory neurons produce neuropeptides including substance P, CGRP, VIP, and corticotropin-releasing hormone, which promote mast cell activation in a positive feedback loop.^70^ Mast cell degranulation can also be regulated by neurotransmitters from sympathetic and parasympathetic neurons.^70^ Mast cell interactions with neurons have been linked to the pathogenesis of pain and inflammation in food allergy and IBS.^48,70,71^

The vagus nerve mediates sensory and parasympathetic regulation of gut physiology. In an ovalbumin (OVA) induced food allergy model, vagus nerve stimulation limits the severity of allergic responses, potentially through inhibition of mast cell expansion.^72^ By contrast, vagotomy does not affect development of OVA-mediated allergy. One possible mechanism of this neural-mast cell regulation involves sensory neuron production of CGRP, which suppresses mast cell degranulation and expansion.^73^

Nerve-mast cell communication is also thought to be central for the progression of IBS, based in part on the observation of mast cell expansion near mucosal nerves in IBS patient colons.^74^ Nerve-adjacent mast cells are associated with the severity and frequency of abdominal pain in IBS patients.^74^ Both mast cells and enteric neurons can produce VIP, which promotes mast cell degranulation and progression of IBS (Fig. 2d).^71^ Recent work indicates that IBS patients have higher systemic VIP levels, more VIPR2-expressing mast cells in the colon, and increased bacterial translocation into the tissue.^75^ Treatment of biopsy samples with VIPR2 inhibitors limits mast cell degranulation and prevents bacterial translocation, suggesting that VIP-mast cell signaling maintains the mucosal barrier during IBS.

While these studies suggest a major role for a nerve-mast cell axis in the development of inflammatory gastrointestinal disorders, a better mechanistic underpinning of this communication is needed using targeted genetic or pharmacological studies in animal models. The heterogeneity of neurons and mucosal mast cells adds another layer of complexity to this system, and future work will be required to identify the neuronal populations and mast cell subsets involved in this process. Finally, it will be necessary to examine the role of this bi-directional communication in other intestinal inflammatory diseases.

### Neuron–ILC interactions

**Fig. 3:**
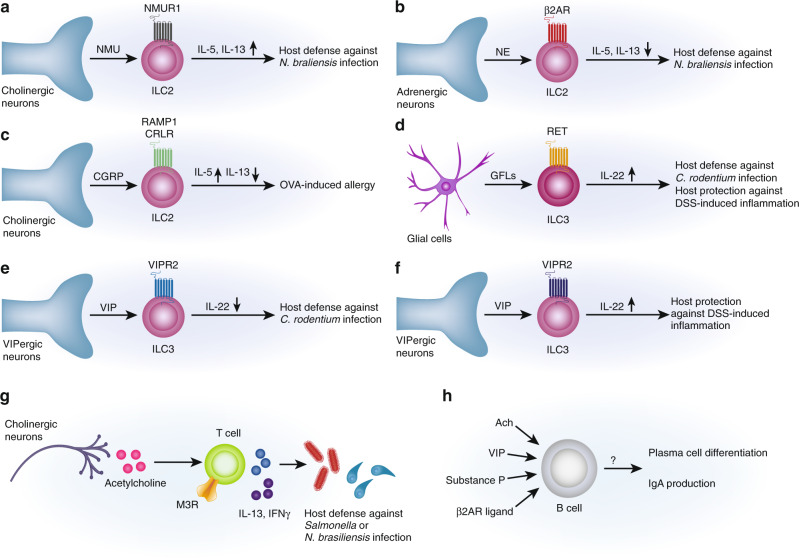
Neuronal crosstalk with intestinal innate and adaptive lymphocytes. **A** Neuronal production of NMU promotes NMUR-dependent ILC2 production of IL-5 and IL-13, which mediate host defense against *N. braliensis* infection. **B, C** Neuronal norepinephrine and CGRP release limits IL-5 and IL-13 production by ILC2s, suppressing host defense against *N. braliensis* infection. **D** GDNF-family ligands (GFLs) released by enteric glial cells promote IL-22 production by RET-expressing ILC3s, promoting host defense against *C. rodentium* infection and host protection against DSS colitis-induced inflammation. **E** VIP production by VIP+ enteric neurons suppresses IL-22 production by ILC3s in a VIPR2-dependent manner, suppressing host defense against *C. rodentium* infection. **F** VIP production by VIP+ enteric neurons enhances IL-22 production by VIPR2-expressing ILC3s, mediating host protection during DSS colitis. **G** Cholinergic neuron production of acetylcholine mediates T cell production of IL-13 and IFNγ, which are crucial for host defense against intestinal *Salmonella* and *N. braliensis* infections. **H** Neurotransmitters and neuropeptides such as acetylcholine, VIP, substance P, and β2AR agonists regulate B cell differentiation into plasma cells and resulting production of immunoglobulins.

ILCs are barrier resident lymphocytes that play critical roles as first responders to tissue damage, actively mediating both host defense and immune responses. Recent work has shown a major role for neuron–ILC crosstalk in the gut and other barrier tissues. Neurons actively crosstalk with both type II ILCs (ILC2s) and type III cells (ILC3s) to regulate gut homeostasis and pathogen infection. Cholinergic enteric neurons expressing the neuropeptide neuromedin U (NMU) play a critical role in host protection during intestinal helminth infection. ILC2s express high levels of neuromedin U receptor 1 (Nmur1), which specifically recognizes the NMU (Fig. 3a).^76–78^ NMU treatment activates ILC2s in vitro to promote their proliferation and secretion of type 2 cytokines in a Nmur1-dependent manner, while NMU treatment in vivo initiates protective responses against oral infection with *Nippostrongylus brasiliensis*. ILC2s colocalize with NMU+ neurons, suggesting a potential role for these neurons in ILC-mediated defense. Consistent with this idea, in vitro enteric neuron organoids produce NMU in a MyD88-dependent manner after stimulation with either the alarmin IL-33 or products from *N. braliensis.*^76^ Therefore, NMU is expressed by a subset of enteric neurons, which signals to ILC2s to promote type 2 intestinal immunity.

The neurotransmitter norepinephrine and neuropeptide CGRP also play major roles in modulating ILC2 function. Intestinal ILC2s colocalize with adrenergic nerves in the gut and express β_2_AR.^79^ By contrast with Nmur1, β_2_AR signaling is counterproductive for host defense during *N. braliensis* infection.^80^ β_2_AR deficiency enhances type 2 immune responses and helminth expulsion, while treatment with a β_2_AR agonist impairs this immunity (Fig. 3b). The neuropeptide CGRP, which is expressed by both the ENS and extrinsic sensory neurons, also antagonizes ILC2 expansion and IL-13 expression in intestinal type 2 immunity (Fig. 3c).^80^ Recent work shows that ILC2s also express CGRP during infection, mediating auto-inhibition of IL-13 production, suggesting a negative feedback loop for ILC2 regulation.^80^

ILC3s sense luminal microbial and dietary perturbations in the gut, driving type 3 immunity through secretion of proinflammatory cytokines like IL-22. ILC3s have been shown to be regulated by enteric glial cells, which are abundant cells that signal to enteric neurons through secretion of factors including glial-derived neurotrophic factor (GDNF) family members (Fig. 3d).^81^ It was found that ILC3s also express high levels of the GDNF receptor RET.^82^ While RET signaling has no effect on overall ILC3 development, it facilitates IL-22 production by these cells. RET deficiency leads to a decrease in intestinal IL22-expressing ILC3s and a concomitant decrease in IL-22-dependent epithelial expression of antimicrobial peptides (AMPs). Animals lacking RET signaling are more susceptible to tissue damage caused by DSS colitis or *C. rodentium* infection.^82^ Transcriptomic analysis reveals RET expression by other immune cells including monocytes, T cells, and B cells, suggesting broader immune regulation by GDNF.^81^

In addition to enteric glial cells, a recent study shows that enteric neurons expressing the neuropeptide vasoactive intestinal peptide (VIP) project nerves into the lamina propria in close proximity to ILC3s, which express the VIP receptor VIPR2 (Fig. 3e).^32^ Food consumption stimulates neuronal production of VIP, which inhibits ILC3 production of IL-22 and abrogates intestinal expression of AMPs. Chemogenetic activation of VIP+ neurons leads to a decreased proportion of IL-22^+^ ILC3s and renders the host susceptible to oral *Citrobacter rodentium* infection.^32^ By contrast, a second study shows VIP promotion of ILC3 expansion and IL-22 production (Fig. 3f).^83^ Administration of VIP induces ILC3 IL-22 production, which is abrogated in VIPR2 deficient mice. VIPR2-deficient mice displayed constitutively fewer IL-22^+^ ILC3s, associated with enhanced susceptibility to DSS-induced barrier disruption and inflammation.^83^

The contradictory findings from these two studies might result from distinct experimental approaches. While one study used total VIPR2 knockout mice,^83^ the other used conditional VIPR2 knockout animals in just RORg+ immune cells.^32^ VIP has been reported to indirectly activate enteric glial cells in a VIPR2-independent manner,^84^ which in turn could secrete GDNF family members to activate the RET signaling in ILCs to promote IL-22 production, and thus may play a role in total knockout mice. One study elegantly used chemogenetic control of VIP+ neurons for temporal silencing or activation,^32^ while the other study mainly utilized VIP peptide injection.^83^ The concentrations of neuropeptide and kinetics of neuronal activation could differentially dictate the outcome of signaling, especially given that GPCRs such as VIPR2 are often modulated by internalization kinetics and ligands in a dose-dependent manner. Lastly, the intestinal microbiota and circadian rhythms have recently been reported to regulate ILC3 cells in the gut,^33^ which may also lead to different outcomes observed in the studies.

These studies show that enteric neurons and glial cells intimately interact with ILCs. Broader studies are needed to elucidate the role of microbial and external cues in modulating neuron-ILC communication. Moreover, identification of brain-gut neural circuits that signal to ILCs, and characterization of how these neurons are selectively triggered in various contexts of mucosal immunity, remains to be explored.

## Neuronal crosstalk and adaptive immunity

The adaptive immune system in the gut plays a fundamental role in establishing immune tolerance in the intestine, maintaining barrier integrity, and developing long-lasting memory against harmful pathogens and antigens.^85^ The role that neurons play in shaping adaptive immunity is only beginning to be understood. Here, we focus on our current understanding of neuronal communication with T and B cells.

### Neuron–T cell crosstalk

CD4^+^ and CD8^+^ T cells, central mediators of adaptive immunity, have been found to interact with the peripheral nervous system,^4^ though the role of these interactions in the intestine are less well understood. In the cholinergic anti-inflammatory reflex, vagal efferent nerves signal to the celiac ganglia, which then relays a signal to the spleen via adrenergic nerves, which signal via β2AR to choline acetyltransferase (ChAT)^+^ T cells that produce ACh.^18^ T cell-derived Ach then signals through nicotinic Ach receptors on macrophages, inhibiting TNF production. Recent work describes that this relay is at play in the intestine: ChAT^+^ T cells are recruited to the colon during *C. rodentium* infection, and T cell-specific ChAT deficiency renders mice more susceptible to infection, suggesting a nonredundant role of T cell-derived ACh in host defense.^86^

Acetylcholine can also play important roles in directly modulating T cell function through both muscarinic and nicotinic receptors (Fig. 3g). The M3 muscarinic ACh receptor (M3R) mediates host defense against intestinal *N. brasiliensis* and *Salmonella* Typhimurium infection. This protection is T cell-dependent, as M3R-deficient mice display impaired T cell-mediated immune and memory responses required for protection against both pathogens.^87^ Both ACh and M3R agonists promote T cell production of IL-13 and IFNγ in a M3R-dependent manner, although the intracellular signaling mechanisms underlying this regulation requires further investigation.

In addition to bacterial and helminth infections, viral infections also impact enteric neurons through neuro-immune signaling. Systemic infection by neurotropic flavivirus (Chikingunya, West Nile, and Zika virus) leads to acute intestinal pathology, bowel dilation, and slowed motility.^88^ This process depends on CD8^+^ T cells that cause enteric neuron death. CD8^+^ T cell deficiency ameliorates the enteric neuron death and improves intestinal transit, while transfer of the flavivirus-primed CD8^+^ T cells restores these phenotypes after infection. Dysregulated CD8^+^ T cell responses could also mediate dysmotility in IBD. Research using IBD patient colon specimens has identified CD8^+^ T cell recruitment to the submucosal and myenteric plexuses correlated with extensive enteric neuron death and severe intestinal transit delays.^89^

Microbes likely play a role in neuronal modulation of T cell function. When Th17-inducing or Treg-inducing bacteria are infused into a microfabricated intestine culture system, these bacteria elicit differential effects on enteric neuron gene expression.^90^ The frequency of RORgt^+^ Treg cells negatively correlate with expression of *Tac1*, which encodes the neuropeptides neurokinin A and substance P. *Tac1*-deficient mice exhibit expanded Tregs, whereas mice fed capsaicin, which activates gut sensory neurons to produce substance P, exhibit decreased numbers of Tregs. These data suggest an effect of intestinal microbes in shaping the ENS, which in turn regulates regulatory T cell differentiation.

While these studies shed light on reciprocal communication between neurons and T cells, further data is needed to understand how gut T cell subsets, including Th1 cells, Th2 cells, Tregs, and Th17 cells are regulated by neuronal signals. It remains to be determined if neurons directly signal to T cells to regulate their development and differentiation, or rather signal via an antigen-presenting cell to affect T cell function. Given that sensory and enteric neurons express cytokine receptors for interferons, IL-10, IL-13, and IL-17, it will also be important to explore how T cells signal to neurons to affect neuronal functions including gut motility, visceral pain, and downstream neuro-immune signaling. It will be interesting to explore whether T cell–neuron crosstalk occurs at homeostasis and is dysregulated in IBD, IBS, or other inflammatory contexts.

### Neuron crosstalk with B cells

Upon activation, B cells switch from IgM- to IgA-producing plasma cells to promote responses to microbes and pathogens.^91^ Plasma cell production in the spleen has recently been reported to be modulated by splenic nerves.^92^ Mechanistically, splenic nerves secrete norepinephrine to activate ChAT-expressing T cells, which produce ACh to promote the differentiation of B cells into plasma cells.^92^ In the intestine, several studies have identified B cells apposing mucosal nerves in the intestine and Peyer’s patch, suggesting that neuronal signals may mediate B cell development or function (Fig. 3h).^93,94^ Consistent with this idea, VIP has been demonstrated to induce IgA production from human B cells in vitro.^95^ Substance P can also promote in vitro expansion of immunoglobin-producing B cells.^96^ In addition, adrenergic signaling through β2AR on B cells promotes IL4-dependent immunoglobin production.^97^ Consistent with this finding, depletion of noradrenaline impairs Th2 cell-dependent antibody production.^98^

B cells may also modulate the health of enteric neurons. In experimental autoimmune encephalomyelitis (EAE), mice display impaired colonic motility and altered glial fibrillary acid protein expression in the ENS.^99^ This is likely connected to B cell immunoglobulin production, as serum isolated from patients with multiple sclerosis or EAE mice exhibit enhanced immunoreactivity against ENS neurons and glial cells. Consistent with this idea, B cell-deficient mice with EAE show normal gut motility.

These limited studies suggest that neurons can regulate B-cell dependent humoral immunity. It is currently unknown, however, whether specific neurons directly or indirectly communicate with B cells in the intestine. Moreover, the context in which enteric neurons dictate the differentiation and function of B cells is poorly understood. Considering that the lamina propria and Peyer’s patch are sites where large numbers of B cells reside, it will be important to determine whether enteric neurons signal to B cells either directly or indirectly to regulate production of antibodies, their antigen presentation, or their function.

## Neuronal crosstalk and epithelial immunity

Extrinsic and intrinsic neurons densely innervate the intestinal crypts and villi in close proximity to the epithelium, and intimate crosstalk between neurons and epithelial cells has been well-described.^100^ Until recently, however, the integration of this crosstalk with respect to immunity has not been well understood. Here, we focus on recent studies illustrating neuron communication with goblet cells and Microfold cells, which impact antigen sampling and innate immunity.

**Fig. 4:**
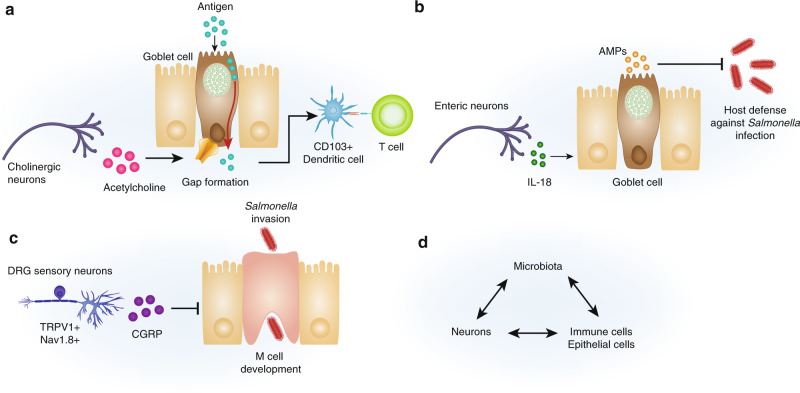
Neuronal crosstalk with intestinal epithelial cells and tuning by the microbiota. **A** Acetylcholine triggers luminal antigen sampling through goblet cell-associated antigen passages (GAPs), resulting in antigen presentation by tissue CD103 + DCs. **B** Neuronal IL-18 regulates goblet cell expression of antimicrobial peptides, which is critical for host protection against intestinal *S*. Typhimurium infection. **C** TRPV1 + Nav1.8+ DRG neuronal release of CGRP suppresses M cell development in the Peyer’s Patch, limiting invasion of *S*. Typhimurium. **D** The host intestinal microbiota tunes neuro-immune crosstalk by impacting neuronal, immune and epithelial function. A healthy microbiota facilitates proper neuro-immune signaling, whereas a dysbiotic microbiota inhibits this signaling.

It is becoming clear that neuron–goblet cell communication plays a crucial role in luminal antigen sampling (Fig. 4a). Small intestine goblet cells sample luminal antigen through goblet cell-associated antigen passages (GAPs), delivering antigen to CD103 + DCs for subsequent antigen presentation to T cells.^101,102^ Recently, GAPs have been implicated in the induction of oral tolerance, and they likely regulate mucosal immunity in other contexts as well.^103^ GAP formation and resulting antigen presentation is mediated by ACh and its neurotransmitter analogs, which act through muscarinic ACh receptor 4 to induce goblet cell secretion and subsequent GAP formation.^102,104^ These data suggest that parasympathetic and cholinergic enteric neurons may critically regulate goblet cell antigen sampling and adaptive immunity in the gut.

Neuron–goblet cell crosstalk is also crucial for the development of innate immunity during enteric infection (Fig. 4b). Recent research indicates that colonic enteric neurons are an important cellular source of the cytokine IL-18, which had previously thought to be expressed exclusively by epithelial cells and immune cells.^105^ Production of IL-18 by enteric neurons, but not epithelial or immune cells, is critical for protection against intestinal *Salmonella* infection. This host defense mechanism relies in part on IL-18 signaling to intestinal goblet cells and promotion of their AMP expression, regulating both mucosa-associated microbiome composition and protection against *Salmonella* infection. This study opens up many new questions, including whether intestinal neurons are major sources of other cytokines, and how neuronal products impact epithelial gene regulation.

Neuron communication with specialized epithelial Microfold (M) cells regulates host defense during enteric infection as well (Fig. 4c). Recent work indicates that CGRP release from DRG nociceptor neurons are critical for protection against *Salmonella* infection.^34^ CGRP suppresses the development of M cells in the ileum Peyer’s patch, which serve as crucial entry portals for *Salmonella* to invade the host.^106,107^ While the finding that neuron-M cell crosstalk regulates host defense has intriguing implications for infection biology, neuropeptide regulation of M cell development has broader consequences for mucosal immunity. M cells mediate critical mucosal immune surveillance by sampling and delivering antigen from the lumen to immune cells for antigen presentation.^108–110^ This function suggests the tantalizing possibility that nociceptor (and potentially other) neuron populations set the immunological tone of the intestine through tuning antigen sampling by epithelial cells.

Until recently, very little has been known about how neuron-epithelial interactions impact immunity and much work remains in this area. The role of neurons in maintaining the gut barrier may be crucial to gastrointestinal biology. While it is clear that neuronal products regulate barrier maintenance through epithelial function, little mechanistic work explains this regulation. Moreover, it is not clear what specific neuron populations interact with epithelial cells or whether other epithelial cell types (like enteroendocrine or Tuft cells) participate in this kind of cross-system communication. Finally, it still remains to be seen what this means for immunity, including critical processes like antigen recognition, generation of adaptive immune responses, and the generation of memory.

## Tuning of neuro-immune crosstalk by the microbiota

The intestinal microbiota is a critical regulator of ENS development and neuron-evoked intestinal function.^42,111^ It is becoming clear that this regulation results from neuronal detection of microbes and initiation of transcriptomic and functional responses.^43,58^ For example, recent work indicates that colonic enteric neurons sense the microbiota through expression of the transcription factor aryl hydrocarbon receptor (Ahr), which responds to a broad array of dietary, microbial, and endogenous metabolites. Neuronal Ahr signaling is dispensable for ENS organization and neuron survival, but is critical for regulation of motility.^42^ Microbes modulate activity of gut-innervating extrinsic neurons as well. Sympathetic neurons show signs of increased c-FOS activity and hyper-activation in germ-free mice, and microbiota colonization limits activation of these neurons through production of the short-chain fatty acid butyrate.^112^

It is not surprising, therefore, that the gut microbiota affects neuro-immune signaling. What is particularly interesting is that microbial signals seem to tune these interactions rather than precipitate or eliminate them (Fig. 4d). While a healthy microbiota promotes proper neuro-immune functioning, dysbiosis leads to abnormal neuron-evoked intestinal function and immunity. This crosstalk is likely reciprocal, as preliminary evidence also suggests neuro-epithelial driven regulation of the composition of the microbiome. Here, we highlight recent molecular examples of microbial tuning of crosstalk between neurons, macrophages and epithelial cells.

A healthy microbiota is critical for maintenance of intestinal neuron-macrophage crosstalk. As described earlier, MMs express BMP2 which signals through BMPR on enteric neurons to drive motility; in turn, enteric neurons produce Csf1 to promote MM survival.^66^ This entire process is tuned by the intestinal microbiota, as antibiotic treatment results in a significant decrease of MM BMP2 expression and a corresponding decrease in the number of activated BMPR+ enteric neurons. Neuronal production of Csf1 is likely modulated by intestinal microbes as well, as microbial-associated molecular patterns including lipopolysaccharides enhance neuronal expression of Csf1 and maintenance of MMs.^66^

Just as a healthy microbiota promotes neuron–MM interactions, microbial dysbiosis can negatively impact neuro-immune crosstalk. For example, enteric infection-induced loss of enteric neurons is directly dependent on microbial dysbiosis resulting from infection.^31^ Treatment with antibiotics following pathogen clearance, and subsequent recolonization with a healthy microbiota, promotes enteric neuron recovery almost to pre-infection levels. Future work will be required to determine how the microbiota modulates neuron–MM signaling to prevent or promote enteric neuron recovery. It is also not clear which commensal species or microbial products are involved; however, ENS recovery is associated with reductions in Bacteroidales and Eryspelotrichaceae spp. suggesting a possible role of these commensals in preventing recovery.^31^

While most research has focused on how intestinal microbes tune neuro-immune and neuro-epithelial interactions, there is evidence to suggest that neurons regulate microbiome composition. 16s rRNA gene sequencing of intestinal microbial communities from Nav1.8+ or TRPV1+ sensory neuron-depleted mice reveals that these populations of neurons regulate the composition of the intestinal microbiota.^34^ Indeed, sensory neuron-depleted mice exhibit significantly less segmented filamentous bacteria (SFB), an epithelial-resident commensal that colonizes the ileum mucosa and lumen as well as the follicle-associated epithelium (FAE) of the Peyer’s patches where M cells reside.^34^ Neuronal maintenance of SFB levels mediates small intestine colonization resistance against *Salmonella* infection, likely through inhibition of pathogen invasion of the FAE.

Consistent with their role in tuning neuro-immune interactions, microbial modulation is reversible such that sequential modulation of the microbiota can reshape signaling.^31,66^ Much work remains to be done to better understand the role of the microbiota in this area, as the examples presented here are likely the tip of the iceberg. It remains to be seen what other neuro-immune interactions are modulated by intestinal microbes. In addition, future work will be necessary to identify the microbes and microbial products that tune these processes to develop broader paradigms for understanding microbe–neuron–immune interactions.

## Future directions

Neuroimmunology in the gut is a nascent field with many exciting research directions. Like a rheostat, the peripheral nervous system potently drives or modulates immunological processes in the gut. The research described in this review article represent recent breakthroughs in our understanding of neuro-immune communication in the intestine, but several important questions are unanswered. How do specific sensory or autonomic neurons and their activity affect gut epithelial and immune cells at the transcriptional, epigenetic, and functional levels?

The recent development of powerful in vivo neurobiology techniques, including optogenetics and chemogenetics, have enabled precise identification and temporal and spatial manipulation of gut-innervating neuron populations in a Cre-dependent manner.^113,114^ Combining these approaches with analysis of changes in immune cell populations using approaches such as single cell transcriptomics or proteomic-based approaches could reveal novel insights into neuro-immune crosstalk. Imaging is another powerful approach to interrogate neuro-immune crosstalk in the gut. Optical clearing techniques have allowed staining of the enteric nervous system throughout different parts of the gut. Adeno-associated virus and pseudorabies virus-based neural tracing techniques can be used to map how distinct intrinsic and extrinsic sensory and sympathetic ganglia, as well as second order brain neurons are affected by immune activation. It would be interesting to determine which neuronal subsets specifically connect with immune cells using these approaches and if they form “synapses” with immune cells. These technological advances will enhance our ability to more carefully map neuronal subsets innervating the gut and determine how they impact immune cells.

Another key remaining question is determining how gut-resident immune cells signal to neurons. GCAMP6-based calcium imaging of enteric neurons and extrinsic sensory neurons can be used in combination with immune stimulation to define whether and how specific gut immune populations including macrophages, T cells, ILCs, and other cell types signal to neurons. The role of neuro-immune communication in several basic aspects gut immunity is still in early stages of investigation—in detection and response to pathogens, antigen presentation, generation of adaptive immune responses, induction of immune tolerance, and the development of immune memory.

Therefore, we envision that the next several years of research will more rigorously use molecular and genetic tools to experimentally characterize and functionally define neuro-immune interactions on both the neuronal and immune/epithelial cell sides of these axes. In terms of immunology, we envision an expansion of our understanding of neuronal communication broadly with many more mucosal immune cell populations than have been studied so far. In other peripheral tissues including the lung, skin, and spleen, neurons have been found to regulate the function of dendritic cells, neutrophils, and γδ T cells. However, little is known about how these cells are regulated by neurons in the intestine. There is even less understood about neuron interactions with other immune and epithelial cell populations including basophils, eosinophils, epithelial stem cells, tuft cells, and Paneth cells.

Our understanding of neuro-immune signaling in disease is also at a very early stage, and we expect this to be a major focus of future research and translation. There are isolated examples of dysregulated neuro-immune circuitry in inflammatory disease contexts such as inflammatory pain in IBD and IBS, however it is currently unclear whether this dysregulation is a direct cause of disease or merely a symptom of tissue inflammation. Whether any of the described or future findings can be translated into therapies for disease remains an important question. There is growing interest in targeted electrical stimulation of nerves such as the vagus nerve (called bioelectric medicine) to treat inflammatory diseases, a strategy which has been reviewed comprehensively elsewhere.^45,115^ In the coming years, we expect to see many more examples of bioelectronic medicine and neuromodulating drugs as promising therapeutic strategies in the intestine to ameliorate IBD, enteric infection, and food allergy.

The nervous and immune systems recognize danger simultaneously and respond in both a simultaneous and coordinated fashion. Both systems involve rapid sensation of environmental cues whose responses regulate tissue and organ function to support healthy physiology. Rather than considering each of these systems and their role in inflammatory disease independently, we posit that these systems should be interrogated and studied as one integrated system. Considering the remarkable number of illustrative recent mechanistic studies exploring neuro-immune signaling, we envision the next several years yielding great strides in our understanding of this integrated system as well as its effect on the generation and maintenance of immunity.
